# Using Machine Learning Algorithms to Estimate the Compressive Property of High Strength Fiber Reinforced Concrete

**DOI:** 10.3390/ma15134450

**Published:** 2022-06-24

**Authors:** Li Dai, Xu Wu, Meirong Zhou, Waqas Ahmad, Mujahid Ali, Mohanad Muayad Sabri Sabri, Abdelatif Salmi, Dina Yehia Zakaria Ewais

**Affiliations:** 1 School of Civil Engineering and Architecture, Nantong Institute of Technology (NIT), Nantong 226000, China; wxshrek@sina.com (X.W.); zmr11@sina.com (M.Z.); 2 Department of Civil Engineering, Abbottabad Campus, COMSATS University Islamabad, Abbottabad 22060, Pakistan; 3 Department of Civil and Environmental Engineering, Universiti Teknologi Petronas, Seri Iskandar 32610, Perak, Malaysia; mujahid_19001704@utp.edu.my; 4 Department of Civil Engineering, Faculty of Engineering, Universiti Malaya, Kuala Lumpur 50603, Malaysia; 5 Peter the Great St. Petersburg Polytechnic University, 195251 St. Petersburg, Russia; mohanad.m.sabri@gmail.com; 6 Department of Civil Engineering, College of Engineering, Prince Sattam Bin Abdulaziz University, AlKharj 16273, Saudi Arabia; a.salmi@psau.edu.sa; 7 Structural Engineering, Faculty of Engineering and Technology, Future University in Egypt, New Cairo City 11835, Egypt; dina.yehya@fue.edu.eg

**Keywords:** steel fiber, concrete, high strength concrete, compressive strength, building material

## Abstract

The low tensile strain capacity and brittle nature of high-strength concrete (HSC) can be improved by incorporating steel fibers into it. Steel fibers’ addition in HSC results in bridging behavior which improves its post-cracking behavior, provides cracks arresting and stresses transfer in concrete. Using machine learning (ML) techniques, concrete properties prediction is an effective solution to conserve construction time and cost. Therefore, sophisticated ML approaches are applied in this study to predict the compressive strength of steel fiber reinforced HSC (SFRHSC). To fulfil this purpose, a standalone ML model called Multiple-Layer Perceptron Neural Network (MLPNN) and ensembled ML algorithms named Bagging and Adaptive Boosting (AdaBoost) were employed in this study. The considered parameters were cement content, fly ash content, slag content, silica fume content, nano-silica content, limestone powder content, sand content, coarse aggregate content, maximum aggregate size, water content, super-plasticizer content, steel fiber content, steel fiber diameter, steel fiber length, and curing time. The application of statistical checks, i.e., root mean square error (RMSE), determination coefficient (R^2^), and mean absolute error (MAE), was also performed for the assessment of algorithms’ performance. The study demonstrated the suitability of the Bagging technique in the prediction of SFRHSC compressive strength. Compared to other models, the Bagging approach was more accurate as it produced higher, i.e., 0.94, R^2^, and lower error values. It was revealed from the SHAP analysis that curing time and super-plasticizer content have the most significant influence on the compressive strength of SFRHSC. The outcomes of this study will be beneficial for researchers in civil engineering for the timely and effective evaluation of SFRHSC compressive strength.

## 1. Introduction

Globally, cement is the most comprehensive construction material due to its easy production, abundant ingredients, and various applications. In its traditional form, concrete is a brittle material with low toughness and lesser strain and energy absorption capacity. Therefore, researchers are exploring ways to reduce concrete’s brittleness and improve its tensile properties. The energy absorption capability of concrete can be enhanced by adding dispersed fibers into it [1,2,3,4,5,6]. Incorporating steel, natural, and synthetic fibers into concrete has been explored in various research studies to enhance its mechanical characteristics, including ductility, fatigue resistance, and crack resistance [7,8,9,10,11,12,13,14,15,16,17,18,19,20,21,22,23]. The toughness and post-cracking behavior of concrete can be improved by the addition of steel fibers [24,25,26,27]. In the construction industry, SFRHSC may be used for pavements, building, repair, rehabilitation, etc. A detailed experimental program needs to be conducted in current practice to determine SFRHSC properties which consume a bulk quantity of time and cost for developing a precise linkage for mix design and its related properties [28]. The variable factors for SFRHSC include cement, fine and coarse aggregates, admixture/super-plasticizer, water, steel fibers, and the type of admixture. An effort was made in the current study to predict the strength parameters of SFRHSC by applying machine learning techniques.

ML approaches are beneficial for solving different multifaceted issues in multiple engineering fields. ML approaches incorporate an input factors database for the outcome prediction. Two ML techniques, a standalone approach based on a single model and Bagging and AdaBoost ensembled algorithms, are employed in this work to predict properties for SFRHSC. The literature depicts that the ensembled ML approach’s performance is preferable to the standalone technique [29,30]. An in-depth evaluation of ML approaches for concrete properties prediction is performed by Chaabene, et al. [31]. In addition to that, the prediction for characteristics of different kinds of concrete, i.e., high-performance concrete (HPC) [32,33,34,35,36,37], self-healing concrete [38], recycled aggregate concrete (RAC) [39,40,41,42], and materials-integrated phase change concrete [43], has been explored. HPC compressive strength estimation was performed with the help of ML techniques by Han, et al. [33]. Water, sand, coarse aggregates, cement, GGBFS, fly-ash, age, and five other variable combinations were input factors. The established model provided an accurate estimation of the compressive strength of HPC. In the current research, ML techniques were applied to estimate the compressive strength of SFRHSC. This study shall be beneficial for researchers to conserve experimental time and cost in the future.

Furthermore, the impact of raw ingredients on SFRHSC compressive strength is not significantly explored yet, indicating a research gap. Therefore, the input parameters/raw ingredients for SFRHSC effect on its predicted compressive strength was also explored and described in this study with the help of a post hoc model-agnostic technique named SHapley Additive exPlanations (SHAP) [20,21]. The ML algorithms SHAP integration was performed to give an understanding of SFRHSC design mix for strength parameter through multifaceted non-linear behavior and the contribution of input parameters are described by allocation of weightage to all input parameters individually. As already mentioned, the precise prediction of concrete types can be made by applying ML techniques. For this purpose, considerable consumption of effort, time, and cost is required in the case of the experimental setup. Hence, it is a need of the hour to establish algorithms based on data modeling and identification of interlinked independent factors and the swift decrease in input matrix dimensions. The employment of ML techniques is important in estimating the behavior of concrete materials. ML techniques application can be claimed as alternative approaches for estimating SFRHSC compressive strength to save experimental time and cost. The application of the standalone ML model and ensembled ML approaches was made in this study. MLPNN is a standalone ML model, whereas Bagging and AdaBoost are ensembled ML algorithms. Additionally, statistical checks were applied for models’ evaluation, and their performances were also compared. Later on, based on the performance of various statistical parameters, a model with accurate SFRHSC prediction was proposed. The explanation of input parameters contribution and ML models’ integration was also made in this study to have a deep insight into mix design for achieving strength of SFRHSC. Overall, a correlation was also developed among interpretable ML approaches and feature importance for considerable properties of the structure.

## 2. Dataset

The database was taken from the literature [44,45] and includes 255 mix designs having 15 input factors with compressive strength of 60–120 MPa. Table 1 exhibits the statistical summary of the database utilized to predict SFRHSC compressive strength. The input parameters include cement content, fly ash content, slag content, silica fume content, nano silica content, limestone powder content, sand content, coarse aggregate content, maximum aggregate size, water content, super-plasticizer content, steel fiber content, steel fiber diameter, steel fiber length, and curing time. The compressive strength prediction variables are based on these input parameters. Anaconda software’s Spyder and Python scripting is employed for SFRHSC compressive strength prediction.

## 3. Machine Learning Approaches

**Figure 1:**
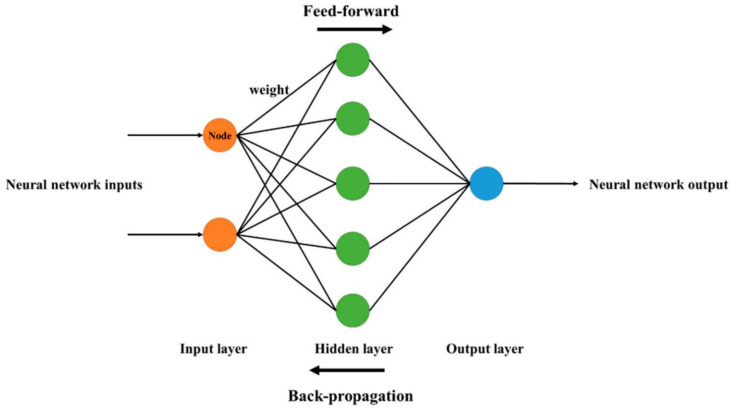
Typical neural network architecture [47].

One of the significant ML models is an approach named artificial neural network (ANN). ANN has a high potential for solving non-linear problems in the environmental and hydrological engineering sectors. The multi-layer perceptron ANN (MLPNN) is the most frequently applied model among different ANN models. There are broadly three layers in the structure of the MLPNN model: i. an input layer, ii. hidden layers (may be one or more), and iii. an output layer. Tansig, purelin, and logsig are three typical MLPNN functions. Its three main and/or important parts are weights, activations, and bias for both hidden and output layers. The weights or model parameters are governed by the models’ training. The tansig activation is applied in hidden layers, and purelin is applied in the case of the output layer. The fivefold cross-validation is adopted to extract the best structure. The three hidden layers (i.e., 9, 3, and 2) are extracted in the top ANN model with the optimum quantum of neurons for each hidden layer [46]. Figure 1 depicts a typical ANN model structure. The network composition is bifurcated into three steps: i. input is processed by a forward pass, ii. multiplication of weight is performed, and iii. model output prediction. The estimated outcomes are then compared with input parameters. Different loss functions are used depending on their performance and objectives. Backward propagation creates back in operation linked individual parameters’ partial derivatives for cost function. The weight of model was updated and propagation of back loss was also performed by utilizing gradient descent.

**Figure 2:**
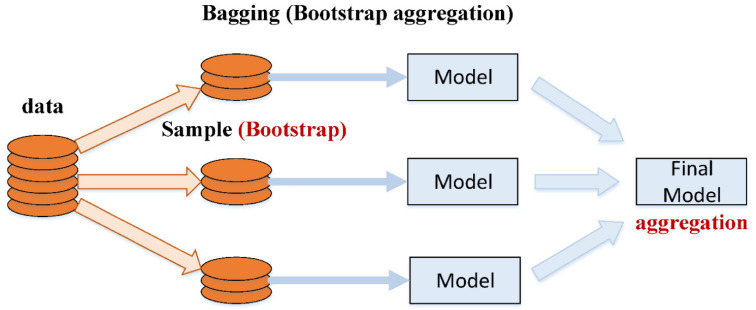
Bagging algorithm procedural flowchart [48].

**Figure 3:**
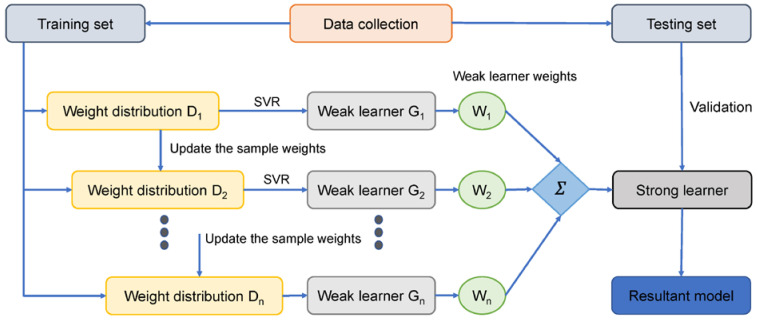
AdaBoost algorithm procedural flowchart [49].

Ensembled techniques may be applied to enhance the recognition and prediction accuracy of ML. These techniques assist in solving over-fitting issues, i.e., sub-model components, by integrating and aggregating different models with weak estimations. The establishment of various sub-models, i.e., A, B, …, N, and training data alteration can generate an intelligent learner. Additionally, the combination measures average and votes are merged to obtain an ideal model with accurate prediction. Bagging is the most widely used ensembled modelling technique, in which the resampling bootstrap approach is employed to gather data and calculate aids. While executing this process, the first training set substitution with partial models was conducted out of the actual model. Few data samples can appear in the number of models; however, some data samples may not appear a single time in the product of any model. The final output of a model is calculated by taking an average of all the model outputs. The Bagging technique, such as the Boosting approach, develops a collective model that develops various components that are more accurate than non-ensembled models. In addition, the Boosting method involves sub-models based on weighted averages to evaluate their addition to the final model. Based on standalone learners such as MLPNN, this study estimates the SFRHSC compressive strength by employing Bagging and AdaBoost techniques. The procedural flowcharts for Bagging and AdaBoost algorithms are shown in Figure 2 and Figure 3.

Moreover, this study recognizes global feature influences and considered feature interactions with SFRHSC, based on a game theory method called SHapley Additive exPlanations (SHAP) [50]. SHAP analysis would increase the proposed model’s explainability. In this approach, each instance prediction is demonstrated by calculating all features, taken for contribution, by employing SHapley values from a coalition of game theory. Each feature value contribution over all the possible amalgamations is slightly averaged for SHapley value. The SHAP values are directly related to the influence of features. The average of each feature SHAP value is taken to achieve the feature influences globally. Later, in terms of importance, the descending order is sorted for these values followed by the plotting of SHAP values. The SHAP value for each feature is depicted from a single point on the SHAP plot. X and Y axis represent SHapley values and feature importance, respectively. Its higher location shows the higher feature influence on SFRHSC on the *y*-axis, and a scale of low to high color is used to depict its importance. The features interaction and their respective influence on SFRHSC are represented from the SHAP plots having a colored scheme to show the feature interaction. This method offers improved information compared to typical partial dependence plots [51]. In SHAP analysis, the importance of feature (*j*) for model output f; $\phi^{j}\left(f\right)$, is the assigned weight for feature contribution summation for outcome of model $f\left(x_{i}\right)$ to obtain probable feature combinations, as a whole [52]. The $\phi^{j}\left(f\right)$ is stated by Equation (1), as presented below:

(1) $$\phi^{j}\left(f\right)=\sum_{S\subseteq \left\{x^{1},\ldots ,x^{p}\right\}/\left\{x^{j}\right\}}\frac{\left|S\right|!\left(p-\left|S\right|-1\right)!}{p!}\left(f\left(S⊔\left\{x^{j}\right\}\right)-f\left(S\right)\right)$$

where

*S* = features subset,
$x_{j}$ = feature *j*, and
*p* = feature number in model.

In this method, a feature’s importance is evaluated by quantifying estimation errors while disturbing a definite feature value. The estimation error sensitivity is considered to assign weight to the feature significance while perturbing its value. SHAP also describes the trained ML model performance. SHAP pays another feature attribution approach, i.e., the linear input parameters addition, to reveal an interpretable model is considered by the model’s outcome. For example, a model having input factors $x_{i}$; where i ranges from 1 to k, and k shows input factor number and *h*($x_{s}$) shows the description model having $x_{s}$ as a simple input; however, Equation (2) is employed to depict an original model f(x):

(2) $$f\left(x\right)=h\left(x_{s}\right)=\emptyset_{0}+\sum_{i=1}^{p}\emptyset_{i}x_{s}^{i}$$

where

p = input feature number and
$\emptyset_{0}$ = constant without any information (i.e., no input).

**Figure 4:**
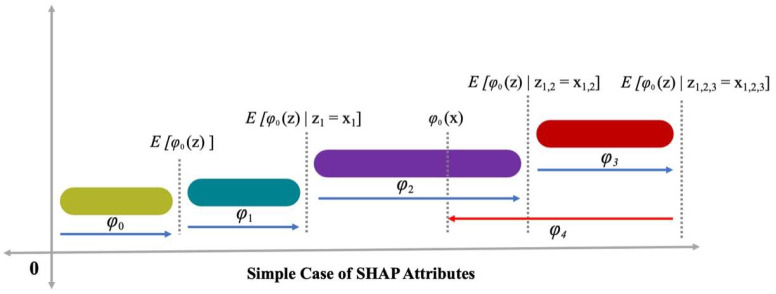
SHAP attributes [54].

$x=m_{x}\left(x_{s}\right)$, i.e., mapping function interlinked with both x and $x_{s}$ input factors. Lundberg and Lee [53] provided Equation (2), in which (*h*()), i.e., the estimation value, was increased by $\emptyset_{0}$, $\emptyset_{1}$, and $\emptyset_{3}$ terms and a reduction of $\emptyset_{4}$ in *h*() value was also detected (Figure 4). A single-value solution to Equation (2), i.e., incorporation of three favorable properties: consistency, local accuracy, and missingness. Consistency confirms no attribution reduction allocated to the corresponding feature in a more influencing feature change. In missingness, it is confirmed to have no important value for missing features, i.e., $\emptyset_{i}=0$ is applied by $x_{s}^{i}=0$. In local precision, it is confirmed that sum-up for attribution of features to be considered as an output function which comprises a model requirement for matching output f for $x_{s}$ as a simplified input. $x=m_{x}x_{s}$ denotes the local accuracy accomplishment.

## 4. Results and Analysis

**Figure 5:**
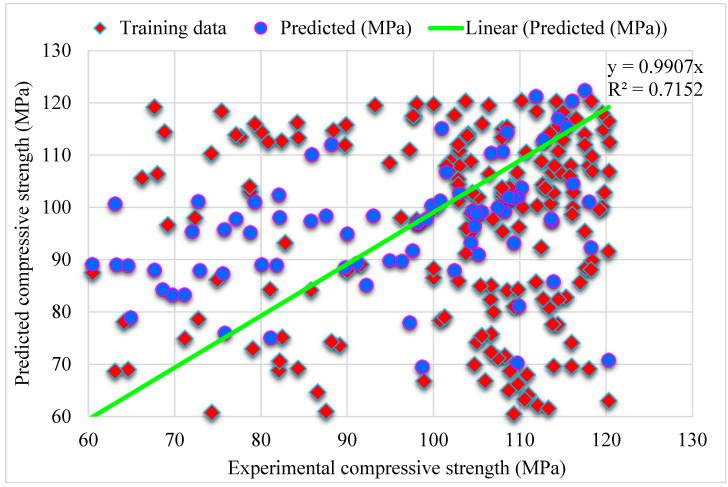
MLPNN predicted and experimental outcomes for compressive strength.

**Figure 6:**
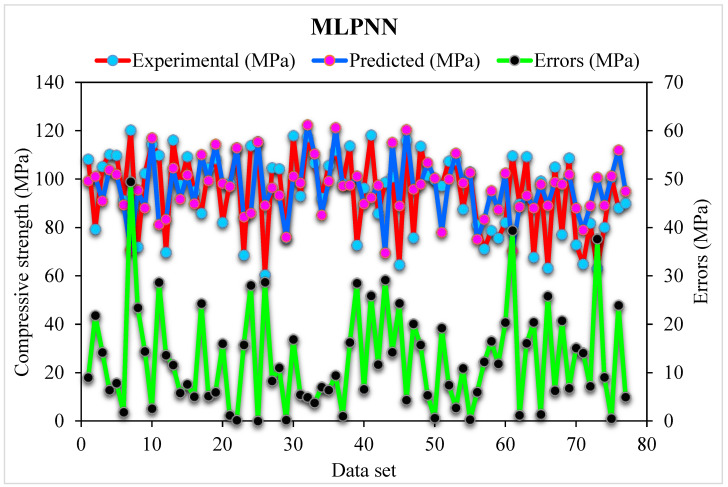
MLPNN predicted and experimental values with errors for compressive strength.

Figure 5 shows the MLPNN predicted and experimental outcomes for SFRHSC compressive strength. The 0.71 R^2^ value demonstrates the least relevant results. At the same time, the estimated outcomes for SFRHSC compressive strength by MLPNN are not in the adequate range. The error distribution of MLPNN predicted, and experimental values for SFRHSC compressive strength are illustrated in Figure 6. Where; 49% of total error values are less than 10 MPa, 26% of these values lie between 10–20 MPa, and 25% are more than 20 MPa.

**Figure 7:**
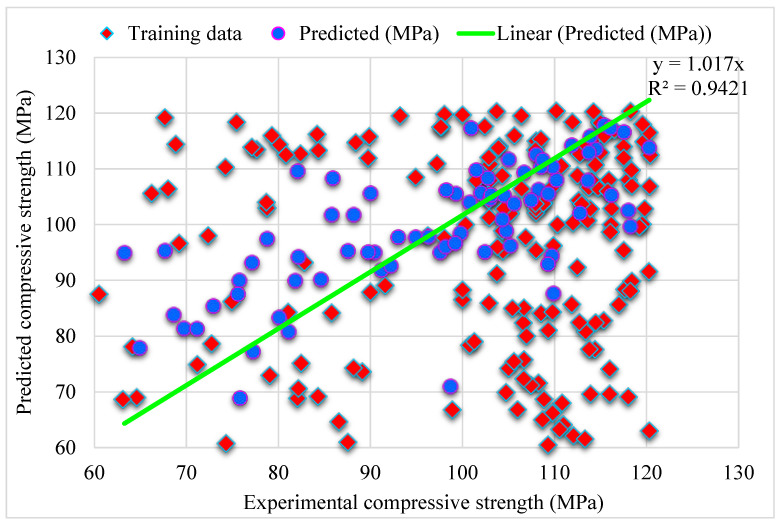
Bagging predicted and experimental results for compressive strength.

**Figure 8:**
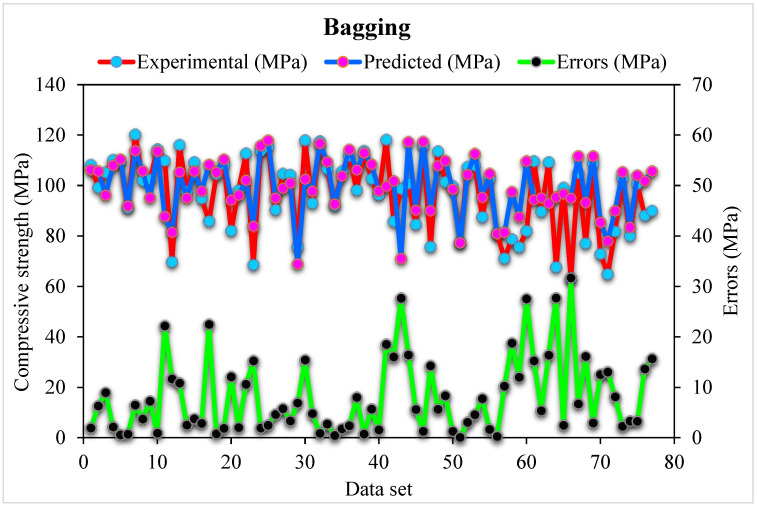
Distribution of Bagging predicted and experimental values with errors for compressive strength.

Figure 7 depicts the predicted Bagging algorithm and experimental outcomes for SFRHSC compressive strength. The 0.94 R^2^ value in the case of Bagging shows high precise results with better accuracy than the standalone MLPNN and Bagging algorithm. The error distribution of Bagging predicted, and experimental values for SFRHSC compressive strength are shown in Figure 8. It is observed that 68% of total error values are less than 10 MPa, 23% of values are between 10–20 MPa, and 9% of values are more than 20 MPa. The higher R^2^ and lower error values depict more precision in the case of the Bagging model than MLPNN. In contrast, the obtained Bagging ensembled ML models’ R^2^ and error values are adequate. Hence, this outcome indicates that Bagging prediction results have higher precision than other models.

**Figure 9:**
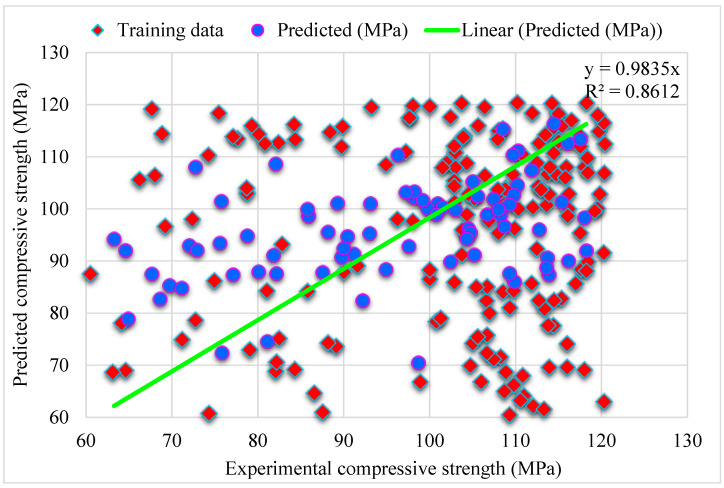
AdaBoost predicted and experimental and results for compressive strength.

**Figure 10:**
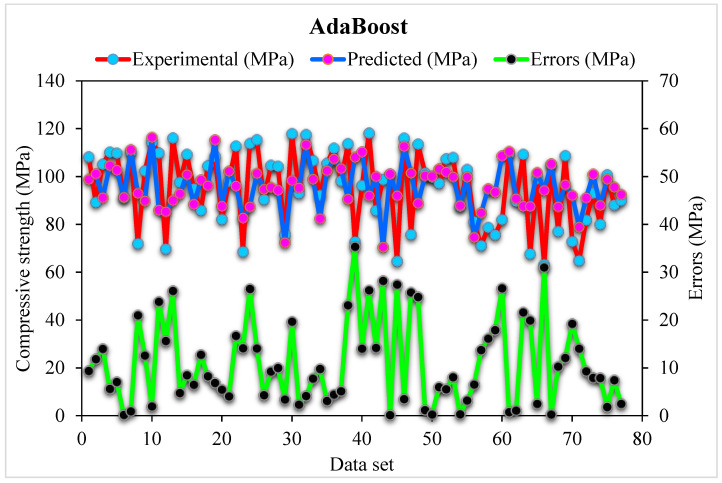
AdaBoost predicted and experimental values with errors for compressive strength.

The AdaBoost algorithm predicted- and experimental values are compared for SFRHSC compressive strength, as shown in Figure 9. The AdaBoost shows less error variance for SFRHSC compressive strength and better-estimated outcomes than that of standalone MLPNN. The adequacy of the AdaBoost model is represented by an acceptable, i.e., 0.86 R^2^ value. The error distribution of AdaBoost predicted and experimental for SFRHSC compressive strength is illustrated in Figure 10. The average error value for SFRHSC compressive strength is 11.16 MPa. Where; 58% of total error values are below 10 MPa, 26% of these values are between 10–20 MPa, and 16% value is more than 20 MPa.

## 5. Discussion

### 5.1. Comparison of All Models

**Figure 11:**
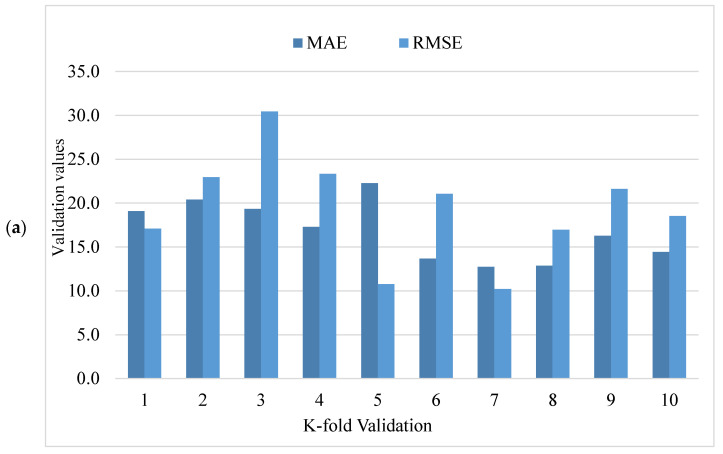

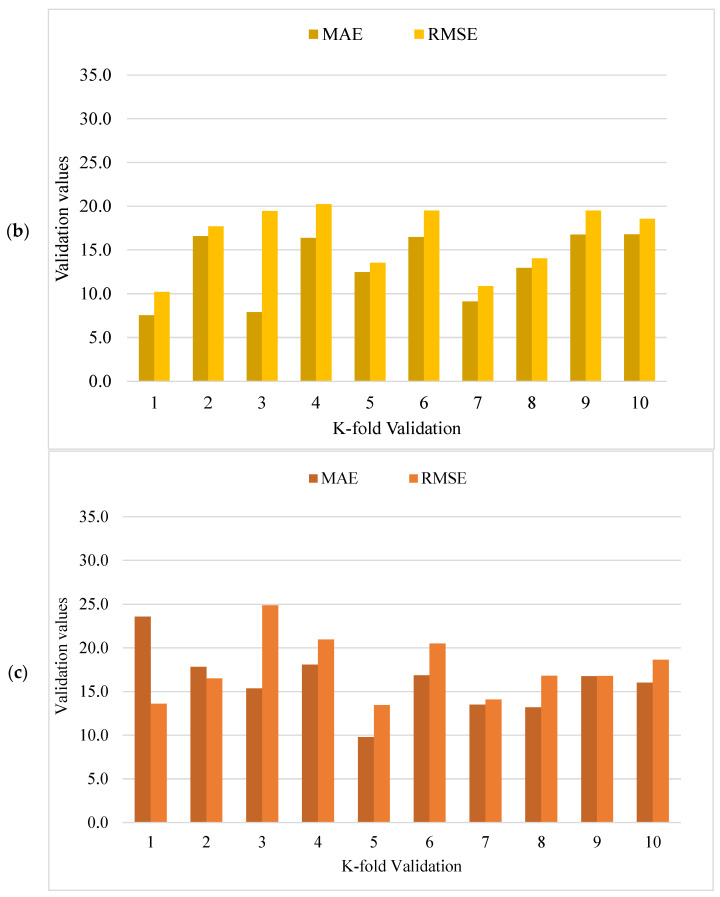
Statistical representation of compressive strength: (**a**) MLPNN; (**b**) Bagging; (**c**) AdaBoost.

During execution, the validity of models was assessed using the k-fold cross-validation approach. The model’s performance was evaluated by applying statistical checks [55,56,57,58]. Generally, in the k-fold cross-validation method, data are split into ten groups for random dispersion with ten times the repetition of this method to achieve satisfactory outcomes. Statistical checks for all the models are listed in Table 2 and Figure 11a–c. The R^2^ values for SFRHSC compressive strength are 0.71, 0.94, and 0.86 in the case of standalone MLPNN, Bagging, and AdaBoost models, respectively, as illustrated in Figure 5, Figure 7 and Figure 9. Table 2 shows the values of MAE and RMSE for SFRHSC compressive strength. The MAE are 12.77, 8.12, and 11.16 in the case of standalone MLPNN, Bagging, and AdaBoost models, respectively. The RMSE are 16.37, 11.06, and 14.22 for MLPNN, Bagging, and AdaBoost models, respectively. It may be noted that the R^2^ value in the case of Bagging is higher compared to other considered models having lesser error values for compressive strength of SFRHSC. A comparison of current model with previous models is shown in Table 3.

**Figure 12:**
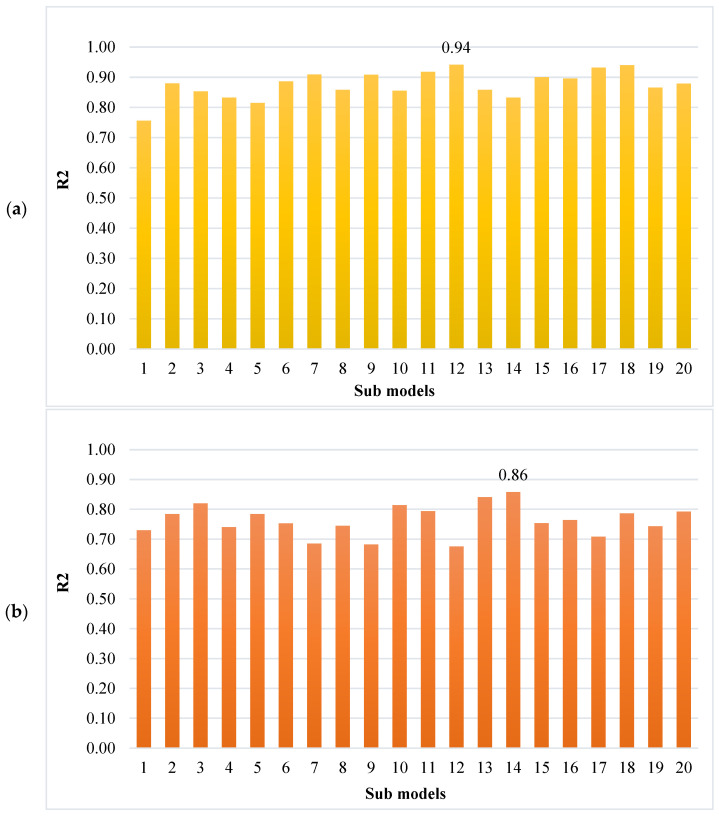
Compressive strength sub-models results: (**a**) Bagging; (**b**) AdaBoost.

To obtain efficient and reliable results, ensembled ML approaches are applied in the current study to predict SFRHSC compressive strength. The Bagging algorithm having a 0.94 R^2^ value offers a more accurate prediction for SFRHSC compressive strength. To predict SFRHSC compressive strength, an optimized model, out of 20 sub-models, is utilized for ensembled Bagging ML models that have better performance (Figure 12a,b). More precision and lesser error are observed in the case of ensembled Bagging models than in other models.

### 5.2. Effect of Input Parameters on the Outcome Using SHAP Analysis

**Figure 13:**
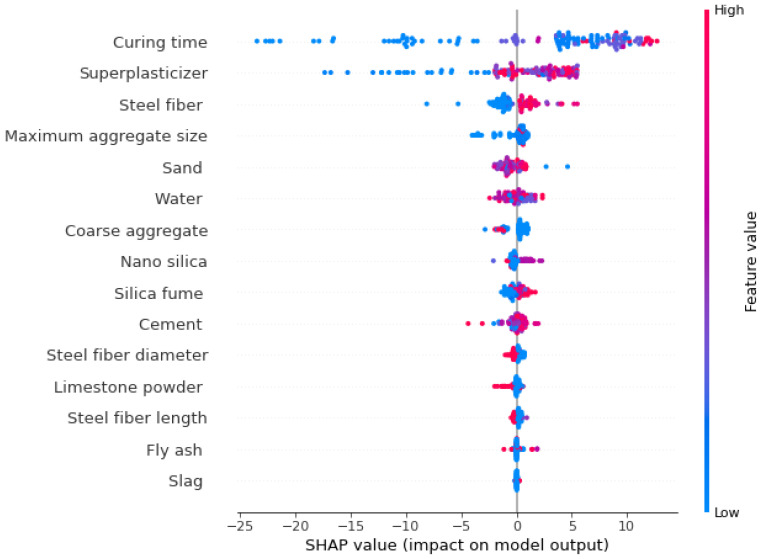
SHAP plot.

The values of each considered feature for SFRHSC compressive strength are plotted in the form of violin SHAP plotting, as illustrated in Figure 13. A different color is assigned for each feature value in this plot, and the respective SHAP value at the *x*-axis shows the contribution outcome. For example, in the case of input features such as curing time and content of super-plasticizer, their positive influence on SFRHSC compressive strength is observed from the right axis. On the rightmost side of the axis, a 14 SHAP value in red points shows that the SFRHSC compressive strength would be higher in enhancing curing time. As far as the super-plasticizer feature is concerned, it may depict a positive influence but only till optimum content. Above optimum content, the negative impact is depicted in the form of blue color points (i.e., lower values). Super-plasticizer is a key parameter for achieving the high strength of concrete by reducing the *w*/*c*. Steel fiber feature also positively influences SFRHSC compressive strength. Then, in the case of maximum aggregate size, it impacts both ways, i.e., positive and negative. However, sand negatively influences SFRHSC compressive strength. As in the case of enhancing sand content, the surface area increases, and ultimately cementitious material would be utilized more in the sand coating. Similarly, the water content feature is a positive influence up to a certain limit, beyond which it would be a negative influence. Down the list, nano silica, silica fume, and cement contents also positively influence the SFRHSC compressive strength.

**Figure 14:**
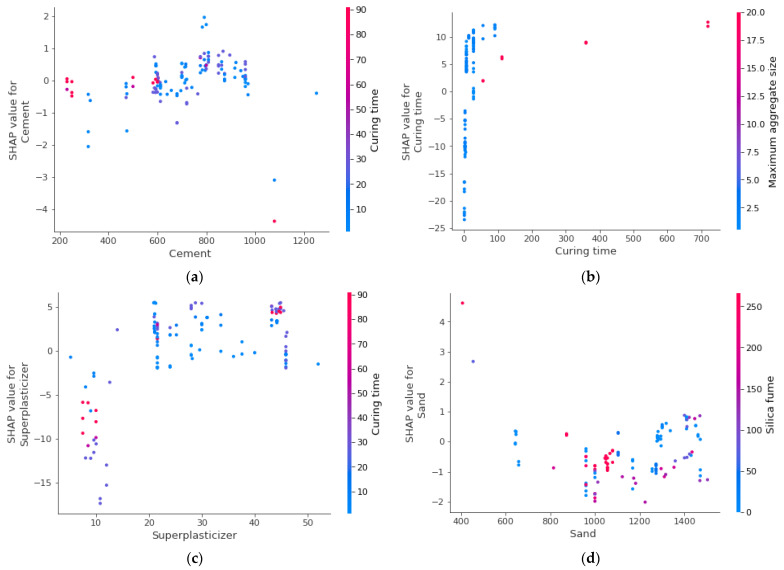

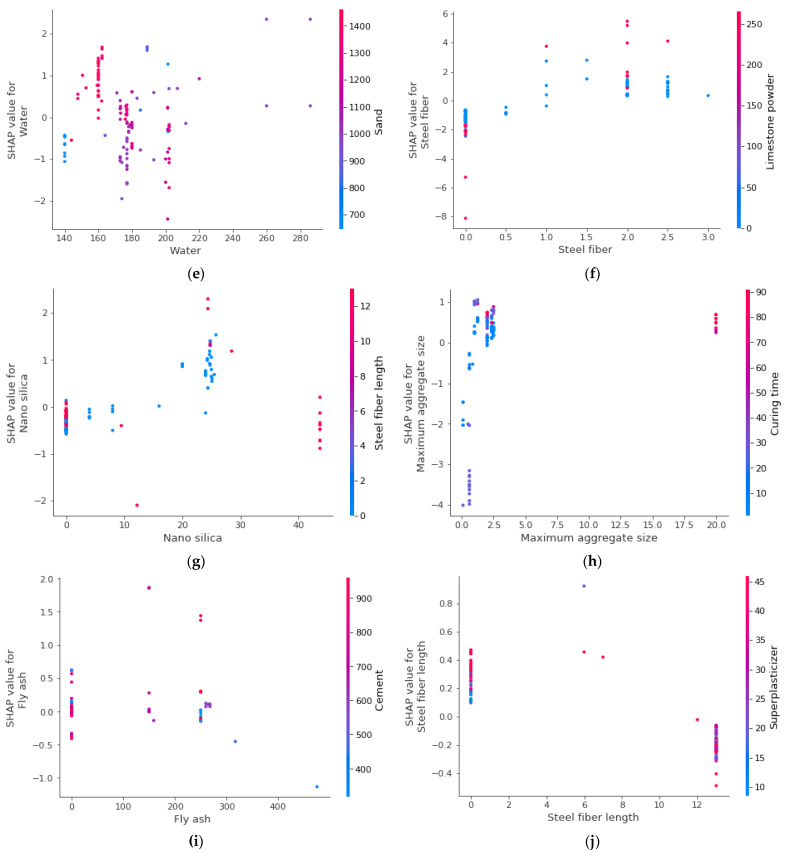
SHAP interaction plot of parameters: (**a**) Cement; (**b**) curing time; (**c**) superplasticizer; (**d**) sand; (**e**) water; (**f**) steel fiber; (**g**) nano silica; (**h**) maximum aggregate size; (**i**) fly ash; (**j**) steel fiber length.

The SHAP interaction plot for all the considered input parameters is shown in Figure 14. The cement content is directly related to SFRHSC compressive strength, and its interaction increases with the curing time. As can be observed from Figure 14a, up to 1000 kg/m^3^ is used as required for HSC. The curing time shows positive linear relation with SFRHSC compressive strength (Figure 14b). Similarly, as presented in Figure 14c, the super-plasticizer also positively influences SFRHSC compressive strength up to optimum content. As depicted in Figure 14d, up to almost 1000 kg/m^3^ content of sand, is showing appropriate influence; however, further addition in its content causes reduction in SFRHSC compressive strength. Water shows both influences (Figure 14e) and its content is kept lesser, as in the case of HSC, and a controlled water content is used with more super-plasticizer content to achieve high strength. As far as the interaction of steel fiber content is concerned (Figure 14f), content up to 2.5% depicts a positive influence; however, beyond this content, its influence becomes negative on SFRHSC compressive strength. Figure 14g shows that nano silica positively influences the SFRHSC compressive strength up to optimum content, i.e., 30 kg/m^3^. The higher content, i.e., 40 kg/m^3^, of nano silica ultimately results in reduced strength due to its larger surface area. Although the aggregate size is a positive influence, the much larger aggregate size may come up with negative results, as shown in Figure 14h. Commonly, a small-size aggregate is used for HSC. The addition of multiple supplementary cementitious materials demands a smaller size of aggregate to achieve higher strength. As in this scenario, the strength of the cementitious matrix is much higher than aggregate strength; therefore, relatively smaller aggregates are preferable for HSC. In the same manner, the fly ash content (Figure 14i) and steel fiber length (Figure 14j) also positively influence it up to an optimum content. As in the case of enhanced steel fiber length, the number of fibers decreases. In the case of compressive strength, the shorter length of fibers is preferable.

## 6. Conclusions

The focus of this research was to evaluate the level of accuracy for machine learning approaches to predict SFRHSC compressive strength. The considered input parameters for said prediction were cement content, fly ash content, slag content, silica fume content, nano silica content, limestone powder content, sand content, coarse aggregate content, maximum aggregate size, water content, super-plasticizer content, steel fiber content, steel fiber diameter, steel fiber length, and curing time. The following conclusions are drawn from current research:

1. The 0.71 and 0.86 R^2^ values standalone MLPNN and ensembled AdaBoost ML models, respectively, demonstrated acceptable outcomes for the compressive strength of SFRHSC. The application of the Bagging approach produced a highly accurate SFRHSC compressive strength prediction from its actual data, which is shown by a 0.94 R^2^ value.
2. Highly effective estimation of SFRHSC compressive strength was observed in the case of the ensembled Bagging model compared to other models. Twenty sub-models ranging from 10 to 200 predictors were used for the optimized prediction of SFRHSC compressive strength.
3. The statistical checks, i.e., RMSE (11.06 MPa) and MAE (8.12 MPa), were employed to determine the model’s performance. At the same time, the larger coefficient of determination and lesser error values depict the better performance of Bagging to estimate the compressive strength of SFRHSC.
4. It is also evident from the k-fold cross validation method upon the comparison of all models that the Bagging model has lower RMSE and MAE and higher R^2^ values for prediction of SFRHSC compressive strength compared to all other models.
5. SHAP analysis reveals that the highest influence is from the curing time on estimating SFRHSC compressive strength, followed by super-plasticizer and steel fiber contents. However, the compressive strength of SFRHSC is least influenced by fly ash and slag. The interaction plot depicts that the cement content positively influences the SFRHSC compressive strength.
6. Among all ML approaches, the Bagging model is the best approach for predicting SFRHSC compressive strength.

This study was based on a wide range of data sets with 15 input variables; however, the database and more input parameters such as workability, specimen size, and curing age need to be generated in future for a better response of the employed models. Users could obtain a much more accurate model by increasing the number of data points/entries, importing a much larger number of mixtures, and considering more input parameters. So, it has been suggested that experimental work, field tests, and numerical analysis, among other things, be used in future studies to increase the number of data points and results (e.g., Monte Carlo simulation).

## Figures and Tables

**Table 1:** Statistical summary of input and output parameters.

		Mean	Standard Error	Median	Mode	Standard Deviation	Range	Minimum	Maximum
Cement content	(kg/m^3^)	719.0	11.7	716.0	960.0	187.1	1021.2	230.0	1251.2
Fly ash content	(kg/m^3^)	47.3	6.2	0.0	0.0	98.8	475.0	0.0	475.0
Slag content	(kg/m^3^)	27.7	6.0	0.0	0.0	95.1	475.0	0.0	475.0
Silica fume content	(kg/m^3^)	94.8	6.3	50.0	0.0	100.5	291.3	0.0	291.3
Nano silica content	(kg/m^3^)	8.2	0.8	0.0	0.0	13.1	43.7	0.0	43.7
Limestone powder content	(kg/m^3^)	67.8	10.6	0.0	0.0	168.9	1058.2	0.0	1058.2
Sand content	(kg/m^3^)	1109.8	17.2	1104.0	960.0	275.1	1095.6	407.8	1503.4
Coarse aggregate content	(kg/m^3^)	90.3	17.5	0.0	0.0	279.9	1162.0	0.0	1162.0
Maximum aggregate size	(mm)	2.9	0.3	2.0	2.0	4.5	19.9	0.1	20.0
Water content	(kg/m^3^)	177.1	1.4	176.9	160.0	22.1	146.0	140.0	286.0
Superplasticizer content	(kg/m^3^)	27.7	0.8	25.2	21.6	13.1	46.9	5.1	52.0
Steel fiber content	(%)	0.9	0.1	0.0	0.0	1.0	3.0	0.0	3.0
Steel fiber diameter	(mm)	0.1	0.0	0.2	0.0	0.1	0.2	0.0	0.2
Steel fiber length	(mm)	6.5	0.4	6.0	0.0	6.4	13.0	0.0	13.0
Curing time	(days)	30.9	5.4	7.0	28.0	87.0	719.0	1.0	720.0
Compressive strength	(MPa)	95.7	1.1	100.0	108.0	17.5	60.0	60.4	120.4

**Table 2:** Statistical checks of MLPNN, Bagging, and AdaBoost model.

Techniques	MAE (MPa)	RMSE (MPa)	R^2^
MLPNN	12.77	16.37	0.71
Bagging	8.12	11.06	0.94
AdaBoost	11.16	14.22	0.86

**Table 3:** ML techniques used in the previous studies and current study.

Ref.	Material Type	Properties Predicted	ML Techniques Employed	No. of Input Parameters	Data Points	Best ML Technique Recommended
[59]	Recycled aggregate concrete	Split-tensile strength	Gene expression programming, artificial neural network, and bagging regressor	9	166	Bagging regressor
[58]	Geopolymer concrete	Compressive strength	Decision tree, bagging regressor, and AdaBoost	9	154	Bagging regressor
[60]	Fly ash-based concrete	Compressive strength	Gene expression programming, artificial neural network, decision tree, and bagging regressor	7	98	Bagging regressor
[61]	Fly ash-based concrete	Compressive strength	Gene expression programming, decision tree, and bagging regressor	8	270	Bagging regressor
Current study	SFRHSC	Compressive strength	MLPNN, Bagging, and AdaBoost	15	255	Bagging regressor

## Data Availability

The data used in this research are properly cited and reported in the main text.
